# Statistics of the Medical Profession in the United States

**Published:** 1872-02

**Authors:** 


### STATISTICS OF THE MEDICAL PROFESSION IN THE UNITED STATES.

Dr. J. M. Toner, Washington, D. C., (*Boston Medical and Surgical Journal*), has completed a synopsis of the list of all the physicians of the United States who have paid the special internal revenue tax of ten dollars on their profession for the year ending April 30, 1871, and forwarded it to W. B. Atkinson, M. D., Permanent Secretary of the American Medical Association, as requested by a resolution of that body, for publication in its forthcoming volume of Transactions. The list, he says, as it is at present, may be considered a complete "Medical Register of the United States."

| | | |
|---|---|---|
| Whole number of physicians of all classes . . , . . | | 49,798 |
| " " of regular physicians, . . . . . | 39,070 | |
| " " of homœopathic " . . . . . . | 2,961 | |
| " " of hydropathic " . . . . . . | 133 | |
| " " of electic " . . . . . . | 2,860 | |
| Miscellaneous and unknown " . . . . . . | 4,774 | |

This gives a ratio of 16.8 physicians to one homœopath in the whole number, and 13.1 regular physicians to one homœopath. Estimating the population of the United States in round numbers at 39,000,000, we have one regular physician to every one thousand of the population. The proportion of homœopathic physicians to the whole population would be about one in every 13,000.

